# Exercise-induced skeletal muscle angiogenesis: impact of age, sex, angiocrines and cellular mediators

**DOI:** 10.1007/s00421-022-05128-6

**Published:** 2023-01-30

**Authors:** Mark Ross, Christopher K. Kargl, Richard Ferguson, Timothy P. Gavin, Ylva Hellsten

**Affiliations:** 1 School of Energy, Geoscience, Infrastructure and Society, Heriot-Watt University, Edinburgh, Scotland UK; 2 Department of Sports Medicine and Nutrition, University of Pittsburgh, Pittsburgh, USA; 3 School of Sport, Exercise and Health Sciences, Loughborough University, Loughborough, UK; 4 Department of Health and Kinesiology, Max E. Wastl Human Performance Laboratory, Purdue University, West Lafayette, USA; 5 Department of Nutrition, Exercise and Sports, University of Copenhagen, Copenhagen, Denmark

**Keywords:** Exercise, Angiogenesis, Capillarisation, VEGF, Endothelial cells, Vascular smooth muscle, Muscle blood flow, Extracellular vesicles

## Abstract

Exercise-induced skeletal muscle angiogenesis is a well-known physiological adaptation that occurs in humans in response to exercise training and can lead to endurance performance benefits, as well as improvements in cardiovascular and skeletal tissue health. An increase in capillary density in skeletal muscle improves diffusive oxygen exchange and waste extraction, and thus greater fatigue resistance, which has application to athletes but also to the general population. Exercise-induced angiogenesis can significantly contribute to improvements in cardiovascular and metabolic health, such as the increase in muscle glucose uptake, important for the prevention of diabetes. Recently, our understanding of the mechanisms by which angiogenesis occurs with exercise has grown substantially. This review will detail the biochemical, cellular and biomechanical signals for exercise-induced skeletal muscle angiogenesis, including recent work on extracellular vesicles and circulating angiogenic cells. In addition, the influence of age, sex, exercise intensity/duration, as well as recent observations with the use of blood flow restricted exercise, will also be discussed in detail. This review will provide academics and practitioners with mechanistic and applied evidence for optimising training interventions to promote physical performance through manipulating capillarisation in skeletal muscle.

## Introduction

Angiogenesis is a key physiological process that occurs to improve oxygen and nutrient delivery to active skeletal muscle. Skeletal muscle angiogenesis is highly regulated by exercise (Gute et al. 1996), and enhanced muscle capillarisation can lead to endurance performance benefits, as well as improvements in cardiovascular and skeletal muscle health. Due to the contribution of capillaries and angiogenesis to skeletal muscle oxygen extraction (for detail on role of capillaries in oxygen flux and uptake in skeletal muscle, please see Pittman et al. 2000), exercise physiologists and rehabilitation specialists are interested in the role angiogenesis plays in exercise performance and cardiometabolic health. Numerous exercise training studies have demonstrated improvements in capillary number in skeletal muscle (Green et al. 1999; Prior et al. 2014; Gliemann et al. 2021), leading to greater oxidative capacity (Murias et al. 2011), $\dot{V}$O_2_max (Liu et al. 2022), and endurance performance (Jensen et al. 2004).

Exercise-induced angiogenesis is a complex process, involving both mechanical and biochemical signals. These are highly coordinated responses, acting together to stimulate angiogenesis. For example, metabolic perturbations can stimulate angiogenic growth factors to be released from skeletal muscle, which can stimulate endothelial cell proliferation (Olfert et al. 2010). Mechanical cues, such as shear and strain, can also regulate the angiogenic response to muscle activity (Wragg et al. 2014; Egginton et al. 2011) via alterations in angiogenic factor release. There is growing evidence for the involvement of other factors, such as lactate (Porporato et al. 2012; Morland et al. 2017) and the involvement of cellular mediators such as pro-angiogenic circulating progenitor and immune cells (Kim et al. 2010), and extracellular vesicles, which have garnered a lot of scientific interest for their ability to stimulate tissue physiological and pathological adaptation (Nie et al. 2019; Nederveen et al. 2021; Zhang et al. 2021).

This review provides an in-depth account of the various mechanisms for exercise-induced angiogenesis, including paracrine regulation, mechanical stimuli, and other cellular contributors (satellite cells, circulating progenitor and immune cells) and critically addresses any sex and age differences in the angiogenic mechanisms and response to exercise. Furthermore, the review will illustrate the impact of exercise duration and intensity on angiogenic signalling and capillary growth, as well as the role of high-intensity interval exercise and other novel modalities, such as blood flow restriction exercise to stimulate angiogenesis. Finally, we provide future directions in the field of exercise and angiogenesis, and the impact these directions could have on our understanding of vessel growth with exercise.

## Exercise-induced angiogenesis

**Fig. 1:**
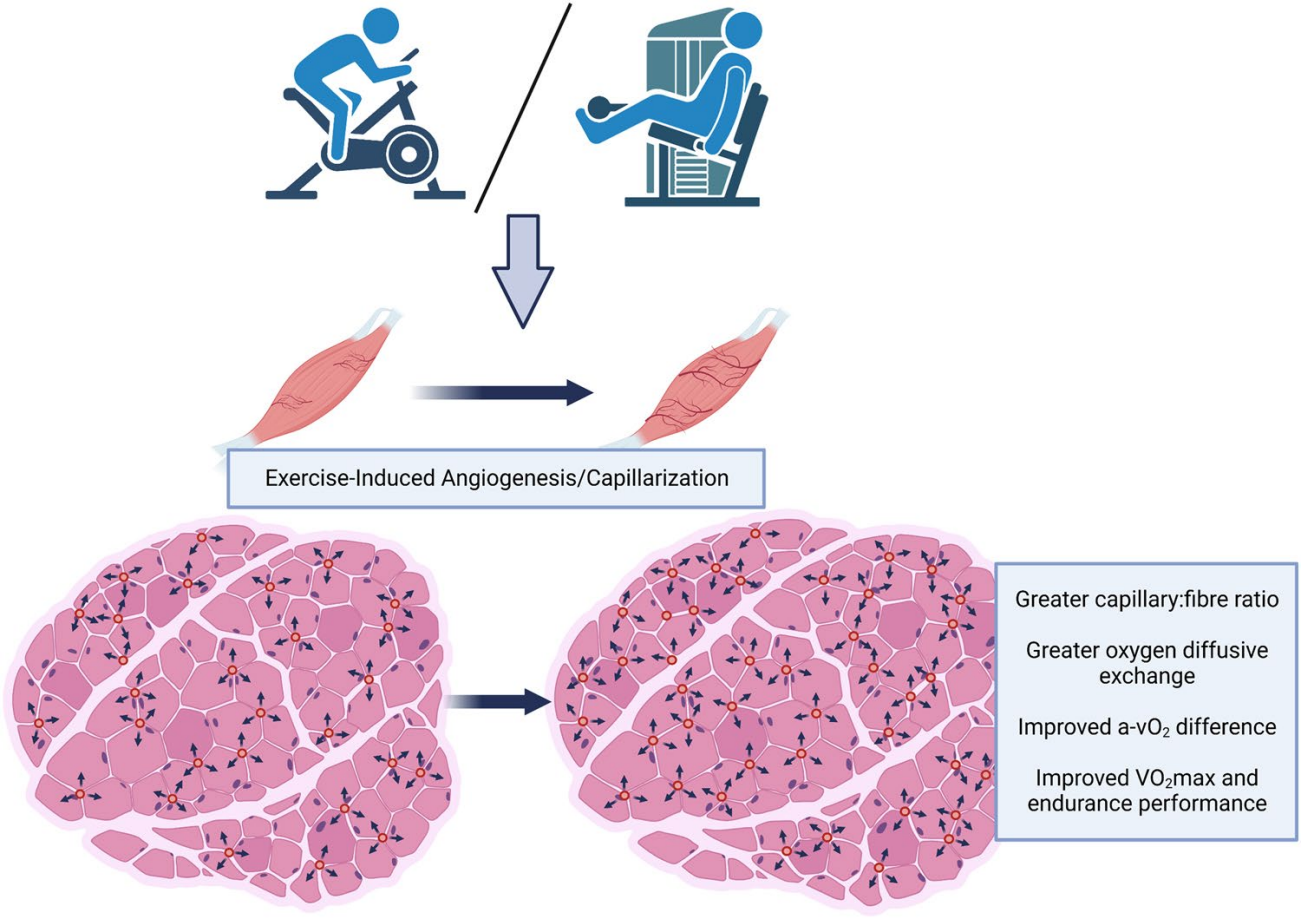
Impact of exercise-induced angiogenesis/capillarisation. Exercise, both endurance and resistance exercise, can lead to improvements in capillary:fibre ratio, resulting in enhanced oxygen diffusive exchange, and thus oxygen uptake ($\dot{V}$O_2_). With reduced capillary-to-fibre distance, nutrients and waste material can be exchanged, leading to improved skeletal muscle performance

Exercise stimulates a wide variety of cardiovascular adaptations, such as improvements in cardiac function (Spence et al. 2013), macrovascular (Landers-Ramos et al. 2016) and microvascular function (Hurley et al. 2019), and angiogenesis (Egginton 2009), the latter of which is the focus of this review. The initial studies describing an increase in capillary density in response to exercise training were performed in the 1960s, with evidence showing exercise trained Sprague–Dawley rats demonstrating a greater capillary density compared to non-exercising rats (Carrow et al. 1967). Since the 1970s, there have been numerous studies detailing the angiogenic response to exercise training in humans (Andersen and Henriksson 1977; Jensen et al. 2004; Huber-Abel et al. 2012; Baum et al. 2015). The improvement in capillary density of skeletal muscle leads to improved oxygen uptake ($\dot{V}$O_2_) and waste extraction, both of which can contribute to enhanced exercise performance (Fig. 1), which is of interest to both athletic and clinical populations. Indeed, capillary density is positively associated with measures of insulin sensitivity (Prior et al. 2014), offering a target for prevention and treatment of chronic diseases such as type 2 diabetes mellitus.

There is a large body of evidence demonstrating a strong link between capillarisation and exercise performance, with measures of capillary density positively associated with training status (Hermansen and Wachtlova 1971; Ingjer 1979a), maximal oxygen uptake ($\dot{V}$O_2_max), ventilatory threshold (Robbins et al. 2009) and critical power (Mitchell et al. 2018). These data suggest that targeting exercise modalities, intensities and durations that can maximise/optimise angiogenesis may be beneficial for improved endurance performance. However, exercise-induced angiogenesis should not be a focus solely for athletic populations but may also prove fruitful for individuals with cardiovascular/cardiometabolic conditions, such as peripheral arterial disease (PAD) (Robbins et al. 2011; Duscha et al. 2020), heart failure (Magnusson et al. 1996; Duscha et al. 1999) and diabetes mellitus (Groen et al. 2014). Studies have demonstrated that exercise can stimulate skeletal muscle angiogenesis in hypertensive individuals (Gliemann et al. 2015a) and augment angiogenic genes in skeletal muscle of patients with HF (Tryfonos et al. 2021), or simply reduce disease-induced capillary rarefaction in those with metabolic syndrome (Frisbee et al. 2006). Together these result in improved oxygen and nutrient diffusion, resulting in improved physical work capacity (Robbins et al. 2011) and metabolic health (Prior et al. 2014).

The improvement in capillary supply in skeletal muscle in response to exercise results in greater diffusive exchange of oxygen (O_2_), nutrients and extraction of metabolic waste compounds (carbon dioxide [CO_2_], ammonia, lactate) due to enhanced surface area of capillaries in skeletal muscle bed and increased erythrocyte transit time. The reduced diffusion distance between capillary and muscle fibre allows for enhanced oxygen supply for oxidative phosphorylation and subsequent ATP re-synthesis, and an augmented metabolic waste extraction which delays the onset of skeletal muscle fatigue. Together, these effects can give rise to improved muscular work rate, or increased time to task failure. However, these effects appear to be local to the working muscle bed whereby the local tissue environment (e.g. hypoxia, growth factor release) and blood flow-related effects (shear stress) are stimulating capillary growth (Badr et al. 2003).

The seminal work by Hudlicka demonstrated that capillary density changes in skeletal muscle in response to electrical stimulation can result from a variety of factors (Hudlicka and Tyler 1984; Hudlicka et al. 1987) including increased blood flow to the muscle causing a mechanical stimulus (shear stress) to the vascular wall (Hudlicka 1991), passive stretch of the tissue induced by muscle contraction (Egginton et al. 2001; Kissane et al. 2021), and metabolism and hypoxia, all of which result in downstream signalling promoting angiogenesis. Such biochemical factors reported to be potential contributors to the exercise-induced growth of new blood vessels include lactate (Morland et al. 2017) and activation of the hypoxia inducible factor-1α (HIF-1α) pathway (Breen et al. 2008; Fiorenza et al. 2020). However, over the past 60 years, research has discovered many more contributors to the angiogenic response to exercise.

## Mechanisms of exercise-induced angiogenesis

### Paracrine and autocrine regulation of skeletal muscle angiogenesis

A large number of pro-angiogenic and angiostatic compounds have been shown to orchestrate the growth of capillaries, and novel angioregulatory factors are continuously being identified. Exciting new knowledge has also been emerging recently with regard to the influence of factors such as endothelial cell metabolism, extracellular vesicles and miRNA on skeletal muscle angiogenesis. In this section, we briefly cover selected angiogenic compounds, shown to be involved in exercise-induced angiogenesis, and then focus on some of the more novel aspects. For a more detailed description of the many pro- and anti-angiogenic factors in skeletal muscle the reader is referred to excellent previous reviews, (Olfert and Birot 2011; Egginton 2011; Haas and Nwadozi 2015).

#### Angiogenic compounds

The most well described and central pro-angiogenic factor in skeletal muscle is vascular endothelial growth factor A (VEGF), which influences several steps in angiogenesis including activation, proliferation and migration of endothelial cells (Ferrara 1999). VEGF protein is present in several different cells in the muscle, including skeletal muscle myofibers, pericytes (Hoier et al. 2013b) and endothelial cells (Milkiewicz et al. 2001) and can exert both paracrine and autocrine effects. Evidence points to a central paracrine role of VEGF in muscle myofibers, as myofibers contain vesicular stores of VEGF and release large amounts of VEGF in response to muscle contractions (Höffner et al. 2003; Høier et al. 2010a; Gavin et al. 2015). Moreover, studies in genetically modified mice, show that myocyte VEGF is important both for basal capillarisation and exercise-training-induced capillary growth (Wagner et al. 2006; Olfert et al. 2010).

At the transcriptional level, VEGF is induced by hypoxia inducible factor (HIF)-1α and estrogen-related receptor α (ERRα) via peroxisome proliferator-activated receptor gamma coactivator (PGC)-1β and PGC-1α (Tang et al. 2004; Chinsomboon et al. 2009; Rowe et al. 2011). These are transcription factors which also regulate other oxidative aspects including mitochondrial biosynthesis (Wu et al. 1999), suggesting a coordinated metabolically coupled regulation. In addition, nitric oxide (NO) is a known regulator of VEGF, involved in exercise-induced (Gavin et al. 2000) and shear-stress-induced (Baum et al. 2004; Williams et al. 2006b) upregulation of VEGF. Nitric oxide can be formed by endothelial NO synthase (eNOS), located primarily in the endothelium, or neuronal NO synthase (nNOS) located primarily in the muscle fibres (Frandsen et al. 1996). Both sources may contribute; however, genetic modification of mice has shown that shear-stress-induced VEGF release and capillary growth primarily involve eNOS (Baum et al. 2004).

Although direct evidence is scarce, capillary growth in response to exercise is thought to proceed both by sprouting, whereby a new capillary grows out of an existing capillary, and by longitudinal splitting, whereby the lumen of a capillary is split into two parallel capillaries (Egginton et al. 2001). Longitudinal splitting occurs primarily by stretching of endothelial cells and requires limited proliferation, whereas sprouting requires endothelial cell proliferation, basement membrane degradation and reconstruction. Consequently, the two angiogenesis forms are associated with different protein expressions (Williams et al. 2006c), whereas both forms involve an upregulation of VEGF and the VEGF receptor Flk-1 (VEGF receptor 2: VEGFR2); the basement membrane alteration required with sprouting angiogenesis is associated with an upregulation of matrix metalloproteinases (MMPs) (Williams et al. 2006c). The finding that exercise upregulates MMP 2 in rodents (Haas et al. 2000; Rivilis et al. 2002) and MMP-9 in humans (Rullman et al. 2007; Hoier et al. 2012) provides indirect support that exercise-induced capillary growth occurs at least in part, via sprouting. In addition, angiopoietin 1 (Ang-1) and 2 (Ang-2), which both compete for the receptor Tie-1, are involved in the breakdown and build-up of the basement membrane and an increased ratio of Ang-1 to Ang-2 has been shown with exercise (Lloyd et al. 2003; Hoier et al. 2012).

Precise regulation of capillary growth is essential and factors inhibiting or modulating angiogenesis, such as thrombospondin-1 (TSP-1), which directly opposes the effect of VEGF, and tissue inhibitor of matrix metalloproteinases (TIMP), which inhibits matrix metalloproteinases, are also upregulated in response to exercise (Kivelä et al. 2008; Olfert et al. 2009; Hoier et al. 2012). The large number of angiogenic compounds described to be involved in capillary growth (Haas and Nwadozi 2015) illustrate the complexity of the process and although much is understood, precisely how the different factors contribute and interact remain to be elucidated.

#### Endothelial cell metabolism

Over the last decade, a role for endothelial metabolism in angiogenic regulation has evolved (for a detailed review see Falkenberg et al. (2019)). Endothelial cells have small amounts of mitochondria compared to, for example, cardiac and skeletal muscle myocytes (Oldendorf and Brown 1975; Park et al. 2014), and the energy contribution from oxidative metabolism is limited. Instead, endothelial cells depend primarily on glycolysis for energy and during angiogenesis, endothelial cells are metabolically reprogrammed towards enhanced glycolysis (de Bock et al. 2013). Consequently, inhibition of glycolysis is shown to impair angiogenesis (Schoors et al. 2013; de Bock et al. 2013). The dependence on glycolysis for energy makes intuitive sense in conditions where capillary growth occurs in response to tissue hypoxia, although maybe less so in response to increased shear stress, when oxygen supply to endothelial cells should be ample. Despite its minor importance as an energy source, endothelial mitochondria are important in angiogenesis as they maintain the NAD+/NADH ratio and provide building blocks for cell growth (Huang et al. 2017; Falkenberg et al. 2019). Accordingly, inhibition of mitochondrial respiration also reduces proliferation of endothelial cells (Diebold et al. 2019; Olsen et al. 2020).

The role of endothelial metabolism may be related to the energy required for proliferation but may also be due to their role in the production of compounds. One such compound, produced by glycolysis, is lactate. Support for lactate in angiogenesis has been provided in wound healing and cancer (Porporato et al. 2012; Brown and Ganapathy 2020) as well as ischemic skeletal muscle (Zhang et al. 2020). In addition to this autocrine effect of lactate, lactate may also originate from paracrine sources. The vast potential of skeletal muscle to produce and release lactate implies that lactate released by muscle fibres during contraction could promote angiogenesis; however, further studies in this area are required.

#### Skeletal muscle-derived extracellular vesicles

Recently, skeletal muscle-derived extracellular vesicles (SkM-EVs) have emerged as potential regulators of exercise adaptations (Choi et al. 2016; Whitham et al. 2018; Vechetti et al. 2021; Murach et al. 2021). EVs are small-membrane-bound signalling vesicles that transfer nucleic acids, protein and lipids to neighbouring and distant cells. Following acute exercise, there is an increase in circulating EVs (reviewed in Nederveen et al. (2021) and Vechetti et al. (2021)), with the implication being that skeletal muscle EVs are released into the circulation to mediate systemic adaptations to exercise. Whilst exercise increases SkM-EV release (Gao et al. 2021), evidence suggests that the exercise-induced increase in circulating EVs are primarily from circulation associated cells and not skeletal muscle (Bryl-Górecka et al. 2018; Brahmer et al. 2019). Indeed, skeletal muscle-specific EV labelling has demonstrated that SkM-EVs make up roughly ~ 1–5% of circulating EVs following acute exercise (Gao et al. 2021; Estrada et al. 2022) and the majority of SkM-EVs likely remain in muscle (Estrada et al. 2022). As EV contents generally reflect the state of their origin cells (Abels and Breakefield 2016), SkM-EVs that remain in muscle following exercise may contribute to local adaptations.

Skeletal muscle tissue capillarisation is proportional to SkM-EV release in mice (Nie et al. 2019; Estrada et al. 2022) suggesting that EVs may be involved in regulating capillarisation. Capillaries are logical targets of SkM-EVs, due to their proximity to muscle fibres (~ 5 μm (Chiristov et al. 2007); however, the angiogenic properties of SkM-EVs at rest and following exercise are not well understood. C2C12 myotubes release EVs that are enriched in several pro-angiogenic miRNAs, compared to endogenous myotube expression (Nie et al. 2019). Additionally, C2C12 myotube-EVs enhance angiogenesis in vitro, through VEGF-independent pathways (Nie et al. 2019). In mice, EVs released by myogenic cells are taken in by endothelial cells following a supraphysiological hypertrophy stimulus (Murach et al. 2021). Interestingly, muscle endothelial cells with high EV internalisation had upregulated expression of matrix remodelling genes, suggesting SkM-EVs may actively regulate aspects of angiogenesis (Murach et al. 2021). Whether SkM-EVs regulate similar pathways to facilitate angiogenic adaptations to endurance exercise is not currently known. SkM-EV regulation of capillaries is an emerging field, and future studies should focus on determining where EVs are distributed in muscle and how EV contents are altered following acute and chronic exercise training to help establish whether they act as novel pro-angiogenic factors.

**Fig. 2:**
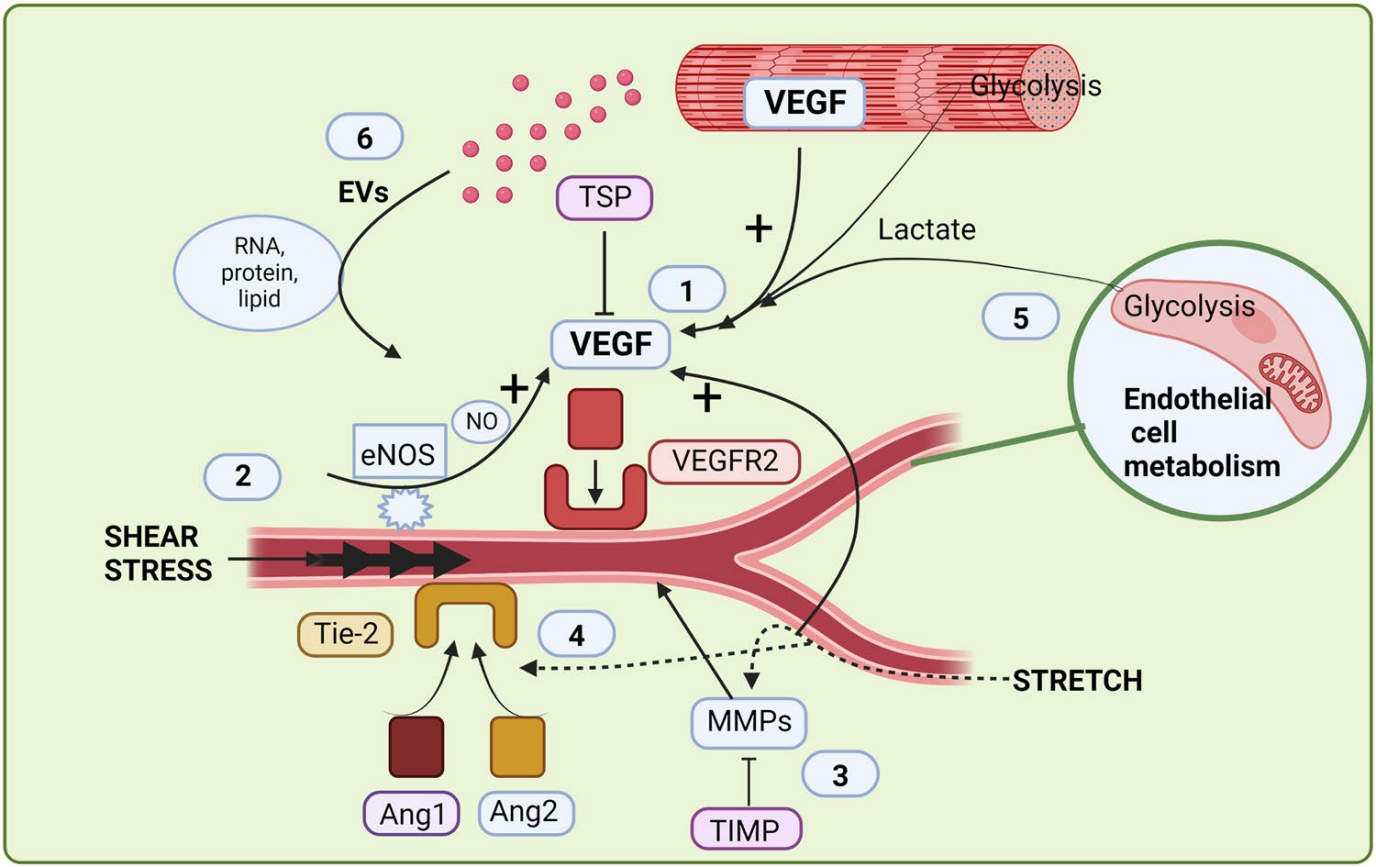
Paracrine and autocrine regulation of skeletal muscle angiogenesis. In skeletal muscle, vascular endothelial growth factor A (VEGF) is a key pro-angiogenic growth factor which induces endothelial cell proliferation and migration by binding to the receptor VEGFR2. VEGF is stored in skeletal muscle fibres and released to the extracellular environment in response to muscle contraction (1). Muscle-contraction-induced angiogenesis is mediated by chemical and mechanical factors, including shear stress and passive stretch. Shear stress leads to capillary growth by longitudinal splitting of capillaries, an effect which in part is induced by increasing nitric oxide (NO) via endothelial NO synthase, in turn upregulating VEGF (2). Passive stretch leads to capillary growth by sprouting, a process which requires modulation of the capillary basement membrane. Important proteins in this modulation are matrix metalloproteinases (MMPs) (3) and angiopoietin (Ang) 1 and 2, acting on the same receptor; Tie-2 (4). Compounds which inhibit angiogenesis, such as tissue inhibitor of matrix metalloproteinases (TIMP; 3) or thrombospondin (TSP; 2), are important for well-controlled capillary growth. Endothelial cells depend highly on glycolysis for energy and angiogenesis is associated with enhanced glycolysis, leading to increased formation of lactate which promotes angiogenesis. Lactate also originates from skeletal muscle fibres (5). Skeletal muscle-derived extracellular vesicles are emerging signalling candidates that may modulate capillary homeostasis and angiogenic adaptations to exercise through paracrine transfer of various angiogenic signalling factors (6)

The contribution of angiocrines and extracellular vesicles on exercise-induced angiogenesis and endothelial cell metabolism is summarised in Fig. 2.

## Cellular mediators—contribution of circulating blood cells to angiogenesis

Skeletal muscle is not the only source of angiogenic factors. Circulating blood cells, including immune cells and progenitor cells, are rich sources of angiocrines, including VEGF (Hur et al. 2004; Gong and Koh 2010; Ruan et al. 2015). The role of progenitor cells (the main focus of the research being endothelial progenitor cells (EPC)) in exercise-induced angiogenesis has been debated but the precise contribution of these cells to the extent of endothelial cell proliferation during angiogenesis caused by exercise is still unknown. These EPCs are widely reported to have vasculogenic capabilities, either through differentiation into mature endothelial cells (Hur et al. 2004; Galasso et al. 2013), or by paracrine action (Hur et al. 2004) similar to that of skeletal muscle discussed earlier in the review. Studies to determine contribution of EPCs to angiogenesis have centred predominantly on tumour angiogenesis, with varying levels of incorporation of EPCs into tumour capillaries (Reyes et al. 2002; Natori et al. 2002; Moccia et al. 2015; Lopes-Coelho et al. 2020). Despite this, exercise studies have been conducted to determine effects on EPC mobilisation into the circulation as a means to ‘home’ these cells to ischaemic regions to support angiogenesis. Single bouts of exercise are known to temporally elevate circulating numbers of EPCs in humans (Rehman et al. 2004; Yang et al. 2007; van Craenenbroeck et al. 2011; Ross et al. 2014, 2018b; Emmons et al. 2016; Montgomery et al. 2019), and long-term training results in sustained improvements in circulating number (Sarto et al. 2007; Manfredini et al. 2009; Schlager et al. 2011; Choi et al. 2014) and improved angiogenic paracrine function (van Craenenbroeck et al. 2010; Sonnenschein et al. 2011).

**Fig. 3:**
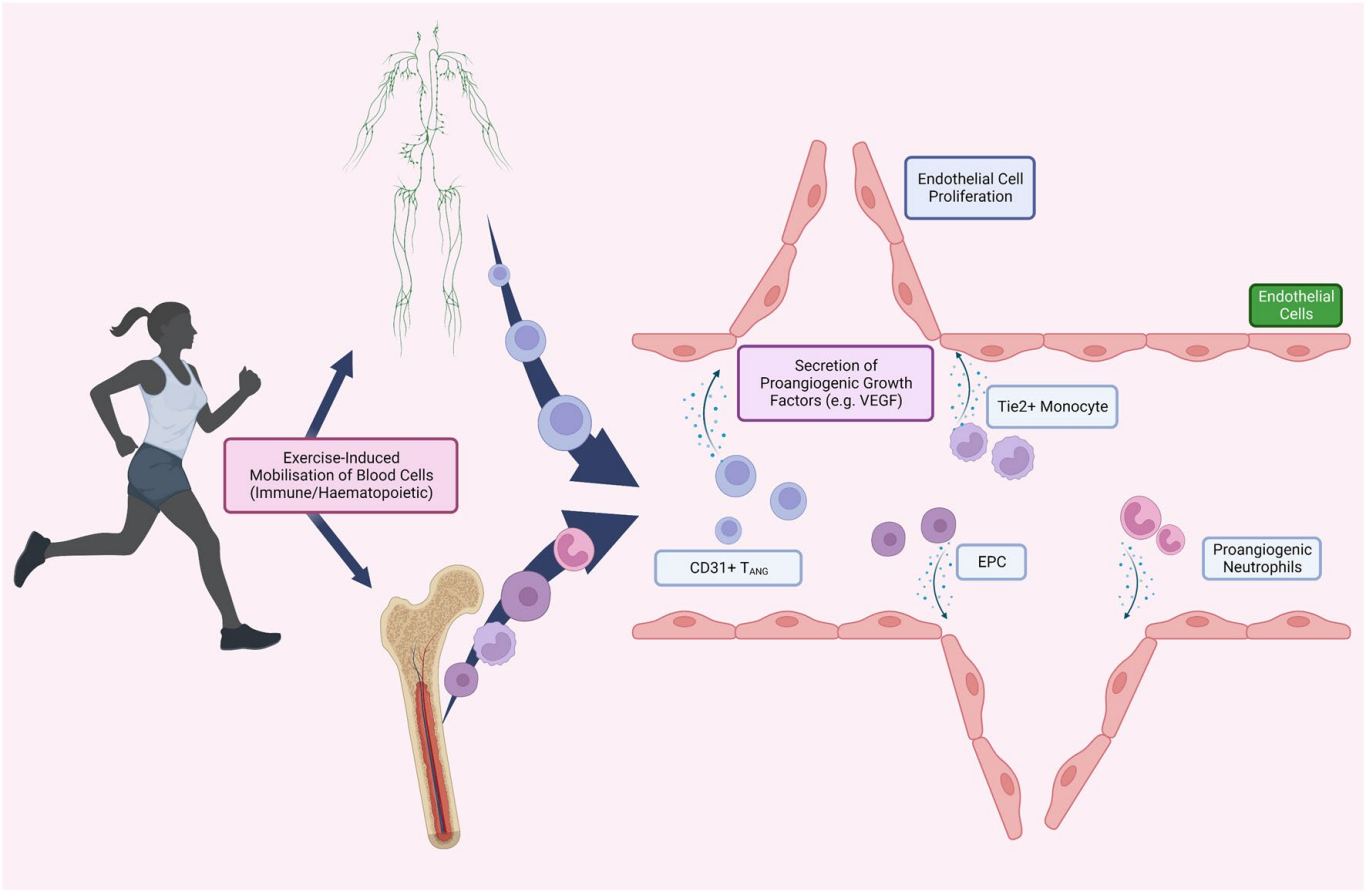
Circulating cells’ contribution to exercise-induced angiogenesis. Exercise can mobilise cells into the circulation from variety of sources, including the bone marrow and lymph tissue. These cells, including endothelial progenitor cells (EPCs) and immune cells (including pro-angiogenic neutrophils, monocytes and angiogenic T cells [T_ANG_]) most likely augment exercise-induced angiogenesis through homing and migrating to ischaemic tissue and subsequent secretion of pro-angiogenic factors, such as vascular endothelial growth factor (VEGF)

Similar to skeletal muscle, EPCs likely stimulate angiogenesis via release of angiocrines packaged in EVs (Mathiyalagan et al. 2017; Ma et al. 2018; Jia et al. 2019; Huang et al. 2022), and there is evidence that exercise can stimulate the release and vasculogenic function of such EPC-EV (Ma et al. 2018). Despite their promise as contributors to exercise-induced angiogenesis of skeletal muscle (Fig. 3), their contribution is still unknown, but given their presence in peripheral blood is rare (< 0.1% of circulating mononuclear cells) (Ross et al. 2018b) and the varying data on their incorporation in tumour vessels, it is likely that their contribution is small, if any. However, readers are directed to two recent reviews detailing the effects of acute and long-term exercise training on EPC counts and function in both diseased (Ferentinos et al. 2022a) and healthy populations (Ferentinos et al. 2022b).

Interestingly, mature immune cells possess significant angiogenic capacity (Freeman et al. 1995; McCourt et al. 1999; Schruefer et al. 2005; Kim et al. 2009; Jaipersad et al. 2014), and in contrast to progenitor cells, are plentiful in the circulation. These include neutrophils (McCourt et al. 1999; Schruefer et al. 2005), monocytes (Kim et al. 2009; Pedragosa et al. 2020) and lymphocytes (Freeman et al. 1995). Studies have identified intracellular pools of VEGF within these leukocytes (Gaudry et al. 1997; Ruan et al. 2015), as well as other important factors required for angiogenesis, such as matrix metalloproteinases (MMPs) (Seignez and Phillipson 2017) offering a potential for regulation of angiogenesis.

Exercise is a significant stimulus for the cellular components of the immune system. Exercise, in a time- and intensity-dependent manner, stimulates the infiltration of leukocytes into the circulation in humans (Rowbottom and Green 2000), with neutrophils and monocyte levels remaining elevated for hours into the recovery period (Robson et al. 1999; Walsh et al. 2011), yet lymphocyte counts reduce significantly within minutes post-exercise (Peake et al. 2017). This lymphopenia is not due to apoptosis, but rather likely due to trafficking of cytotoxic lymphocytes to other tissues, whether part of this lymphopenia may contribute to angiogenesis in ischaemic tissue is unknown, but we do know that a subset of lymphocytes, CD31^+^ T cells, have pro-angiogenic functions (Hur et al. 2007; Kushner et al. 2010), and are mobilised by exercise (Ross et al. 2016, 2018a).

Acute bouts of exercise may also promote angiogenic gene expression in these leukocytes. Neutrophils were found to upregulate platelet-derived growth factor 1 (PDGF-1) and fibroblast growth factor 2 (FGF-2) (Radom-Aizik et al. 2008), both involved in angiogenesis (Li et al. 2003; Presta et al. 2005), and exercise upregulates monocyte chemoattractant protein-1 (MCP-1) levels (Lloyd et al. 2003) which could recruit monocytes into the skeletal muscle to assist with angiogenic processes by secreting VEGF (Ruan et al. 2015)and basic fibroblast growth factor (bFGF) (Arras et al. 1998). Additionally, a subset of monocytes express Tie-2, which acts as a receptor for the angiopoietins, and is involved in regulating angiogenesis (Fagiani and Christofori 2013). In fact, Tie-2-expressing monocytes (TEM) correlate with tumour progression due to their influence on tumour vessel formation (Ribatti 2009; Ji et al. 2013). It has been shown that exercise can acutely increase circulating numbers of these TEMs, which appears to be intensity dependent (O’Carroll et al. 2019); however, studies investigating pro-angiogenic activity are lacking.

Immune cells could play a significant role in exercise-induced angiogenesis (Fig. 3), but despite the research thus far, more mechanistic, relevant studies including both human and animal models are needed to determine whether these leukocytes traffic to exercised muscle and instigate or regulate angiogenesis, and human in vitro studies to determine the paracrine activity in physiologically relevant experiments. Also, these immune cells are likely significantly affected by inflammatory conditions, such as CVD (Poller et al. 2020), kidney disease (Kong et al. 2022), rheumatoid arthritis (Edilova et al. 2021) and diabetes mellitus (Zhou et al. 2018; Lin et al. 2021), in terms of composition of the immune system (see prior references), but also their trafficking (Luster et al. 2005) and wound repair (Moura et al. 2019) functions, but the role of inflammatory conditions to affect immune-mediated skeletal angiogenesis is unknown.

In addition to mature immune cells, platelets, aside from their primary role in the clotting cascade, are also highly pro-angiogenic (Packham et al. 2014; Gliemann et al. 2021). Packham et al. (2014) demonstrated that platelet depletion in a mouse model resulted in abolishment of hyperemic and muscle overload-induced angiogenesis, which was reliant on platelet activation. Platelets do express VEGF (Banks et al. 1998; Gliemann et al. 2021) and act to promote endothelial cell proliferation in vitro (Gliemann et al. 2021). Interestingly, the only study to assess platelet angiogenic capacity in an exercise setting found that activity level (sedentary, low, moderate, high) did not affect platelet-induced endothelial cell proliferation (Gliemann et al. 2021). Acute exercise is a powerful stimulus for platelet activation (Haynes et al. 2018), commonly thought to represent thrombosis risk, and thus may also represent an ‘angiogenic’ activation of platelets.

## (Bio)mechanical cues for exercise-induced angiogenesis

In addition to biochemical signals, the endothelium can also adapt in response to mechanical cues, such as shear, strain and/or stretch. One such mechanical cue associated with exercise is elevated blood flow, resulting in a shear stress stimulus. Studies demonstrated that administration of a vasodilator in both animal models and humans resulted in an expansion of microvascular networks (Rivilis et al. 2002; Williams et al. 2006a; Egginton et al. 2016) which was nitric oxide dependent (Williams et al. 2006a); however, this adaptation was rapidly reversed upon cessation of the stimuli (Egginton et al. 2016). Although these are not exercise studies, they do demonstrate the potential of exercise-induced angiogenesis via shear stress mechanisms. In addition to shear stress, muscle overload is a mechanical cue associated with exercise. Mechanical overload is often induced in animal studies by surgical release of the tibialis anterior tendon, resulting in ‘overload’ of the extensor digitorum longus muscle in mouse models. This mechanical overload muscle resulted in enhanced angiogenesis (Egginton et al. 2011; Tickle et al. 2020; Kissane et al. 2021) via VEGF (Williams et al. 2006c), and improved endurance performance of said muscle (Tickle et al. 2020).

The exact mechanism by which the muscle overload results in angiogenesis is likely multi-faceted, with the contraction and stretch of the muscle during exercise, or overload, resulting in mechanical disruption of the capillary bed, with endothelial cells themselves experiencing tissue stretch, compression and changes in transmural pressure. In fact, there is promise in passive exercise perhaps in individuals with limited exercise capacity, as passive leg movement results in an exercise hyperaemia (McDaniel et al. 2010; Walker et al. 2016) which can result in significant angiogenesis after only 2 weeks of passive limb movement ‘training’ (Høier et al. 2010b). However, such hyperaemia with passive limb exercise is attenuated in older adults (McDaniel et al. 2010) and in PAD patients (Walker et al. 2016); thus, the angiogenic response is also likely to be attenuated.

Such hyperaemia, and thus shear response, is also observed in passive heating of the limbs (Coombs et al. 2021), and data in humans are promising that regular passive heating can result in improved capillarisation of muscle (Hesketh et al. 2019). However, heating of the tissue not only results in angiogenesis due to shear stress, but evidence also suggests that the heating of the limb can result in systemic changes, such as changes in circulating heat shock proteins (Kim et al. 2016; Didier et al. 2022) which may contribute to improvements in endothelial cell proliferation and angiogenesis (Brunt et al. 2019).

## Impact of age and sex on exercise-induced angiogenesis

It is possible that individual characteristics might influence the angiogenic response to exercise. However, before a conversation on the magnitude of the angiogenic response, it is important to recognise the vast range of exercise training with differences in modality (aerobic, resistance, concurrent), intensity (mild, moderate, severe), frequency (days/week), the duration of acute bouts (minutes, hours), and duration of chronic training programmes (weeks, months, years). Also, several studies have investigated differences in muscle capillarisation between well-trained and sedentary individuals where differences may reflect both long-term exercise training and genetics. This spectrum of exercise training makes it difficult to compare results across studies. It can also be difficult to compare values between studies because of differences in the muscles examined and the methods employed to assess capillarisation.

**Fig. 4:**
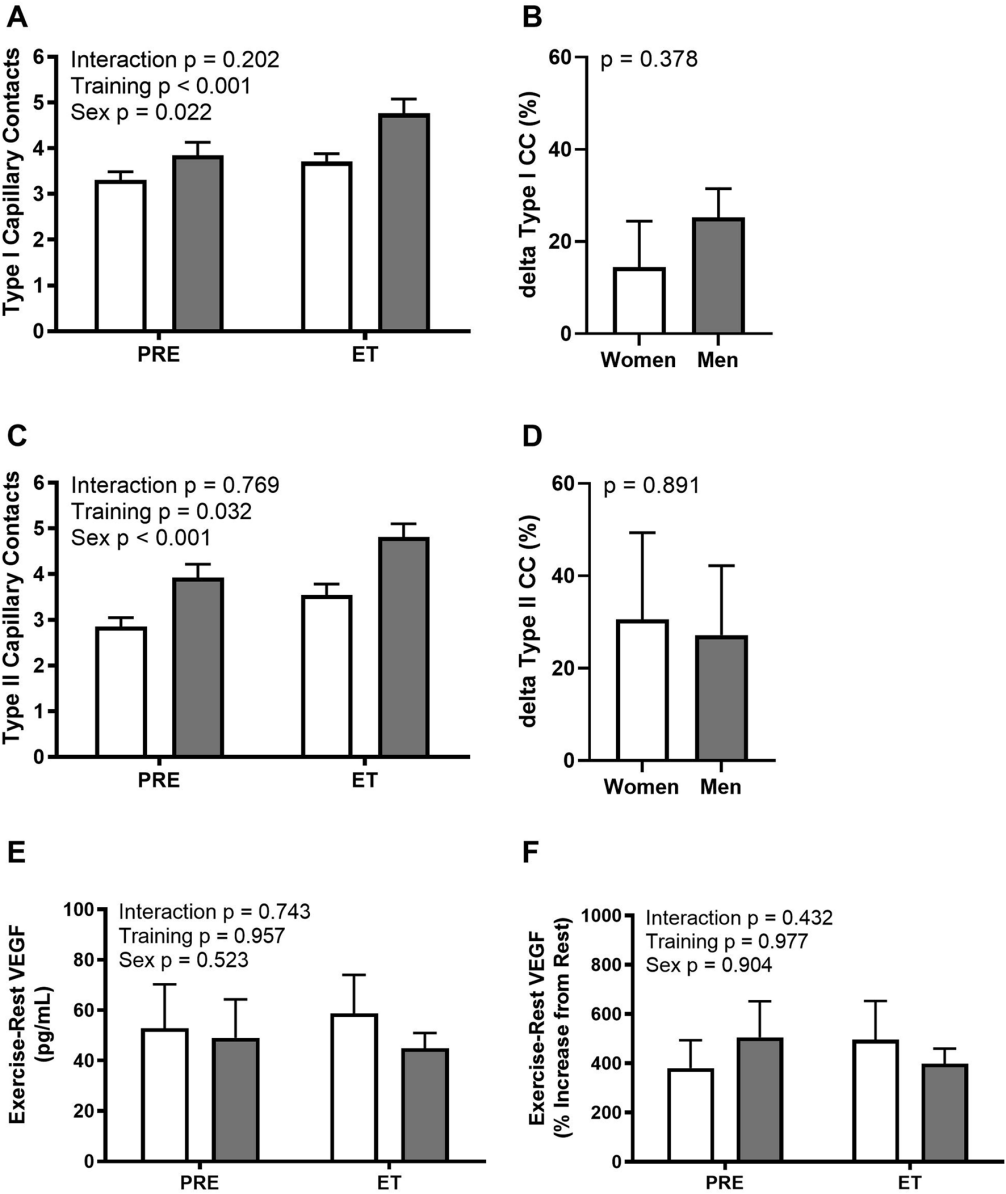
Comparison of the response to aerobic exercise training in females and males for capillary contacts (CC) of type I (**A**) and type II (**C**) fibres; the relative ((post–pre CC)/pre CC × 100)) change (delta) in CC of type I (**B**) and type II (**D**) fibres; and acute exercise-induced increases in absolute (**E**) and relative (**F**) interstitial VEGF. There are no differences in the angiogenic or VEGF responses to aerobic exercise training between females and males. *Pre* before training, *ET* following 8 weeks of aerobic exercise training, *VEGF* vascular endothelial growth factor. *N* = 8/group. Data from Gavin et al. (2015) and (Gavin et al. 2007)

Several studies on aerobic and strength training confirm that angiogenesis occurs in both men and women (Andersen and Henriksson 1977; Ingjer 1979b; Wang et al. 1993; McCall et al. 1996; Green et al. 1999); however, there are only a limited number of studies directly comparing the specific impact of sex on skeletal muscle exercise-induced angiogenesis. Endurance (3 days/week), strength (3 days/week) or strength + endurance (6 days/week) training resulted in 7% (non-significant), 0% and 12% (significant) increases in capillary:fibre ratio, respectively, with no difference between young (mean of 22 years) males and females (Bell et al. 2000). Elastic resistance training increased capillary contacts in young (mean of 20 years) males, but not females (Hostler et al. 2001). To further understand potential sex-based differences in exercise-induced angiogenesis, we reanalysed the angiogenic response from our previously published reports investigating ageing and exercise-induced angiogenesis in females (Gavin et al. 2015) and males (Gavin et al. 2007) (*N* = 6/group) in which an identical exercise training programme design was employed. The reanalysis did not identify any significant differences from pre- to post-training in the absolute change in capillary contacts of type I fibres or type II fibres; or differences in the relative change expressed as a percentile of capillary contacts of type I fibres or type II fibres between females and males (Fig. 4). Consistent with a similar angiogenic responsiveness, there were no differences in the interstitial VEGF response from rest to exercise (exercise − rest) between females (Gavin et al. 2015) and males (Gavin et al. 2007) (*N* = 8/group) either before or after the training programme (Fig. 4). Overall, it appears aerobic and resistance exercise-induced angiogenesis does occur in young males and females, though responses may be different based on the specific mode, duration, frequency and intensity of the exercise stimulus.

In middle-aged individuals (mean age 52 years), 6 months of aerobic training significantly increased capillary:fibre ratio by 31% in females but did not significantly increase capillary:fibre ratio (7.7%) in males (Robbins et al. 2009). Consistent with this, 12 weeks of intense aerobic training increases capillary:fibre ratio ~ 11% in groups of pre- (mean age 49 years) and post-menopausal (mean age 52 years) women suggesting menopause and the associated lack of estrogen do not impair exercise-induced angiogenesis (Pérez-Gómez et al. 2021).

Ageing lowers muscle fibre size and capillarisation of predominantly of type II fibres (Coggan et al. 1992a; Croley et al. 2005; Ryan et al. 2006), which may detrimentally impact women twice as much as men (Coggan et al. 1992a). Over 12 years, aged men (initial age 65 years) lost 20% of muscle capillaries (Frontera et al. 2000). Long-term (9–12 months) aerobic exercise training resulted in similar increases in $\dot{V}$O_2_max, muscle fibre size and capillary:fibre ratio similarly in aged (~ 65 years) men and women (Coggan et al. 1992b). There was no difference in aerobic training-induced increases in $\dot{V}$O_2_max and capillary:fibre ratio between young and aged women (Gavin et al. 2015) and men. However, increases in aged men with intense aerobic training (Gliemann et al. 2014) are not present in similarly aged and trained women (Olsen et al. 2020). It may be that a higher aerobic exercise training intensity may be required in older women (Gliemann et al. 2021).

In older men, resistance exercise can increase capillarisation in conjunction with increases in muscle fibre size (Frontera et al. 1990; Hepple et al. 1996). As with aerobic exercise, there are reports of non-significant changes (+ 20–25%) in capillarisation with resistance exercise in men and women. These findings could reflect either inadequate power due to small sample sizes or a heterogenic angiogenic response in older adults (Ferketich et al. 1998; Hagerman et al. 2000).

To date, potential impairments in age-associated angiogenesis have focussed on the potential role of the VEGF pathway. Acute exercise-induced increases in interstitial VEGF are ~ 50% smaller in aged compared to young men and women (Gavin et al. 2007, 2015). VEGF is 33% lower in myotube-conditioned media in aged compared to young women and the proliferation of primary microvascular cells exposed to growth media is lower in aged compared to young women (Olsen et al. 2020).

## Manipulating exercise variables to optimise angiogenesis

Training-induced physiological adaptations, including capillary growth, are governed by the overall volume of the training, of which the intensity and duration of each exercise session are key determinants. In the athletic endurance-trained population, the overall training volume has been observed to be distributed in a polarised manner (Seiler and Kjerland 2006; Seiler 2010; Stöggl and Sperlich 2015), such that ~ 80% of training is completed at low-to-moderate intensity, whereas the remaining 20% is completed at high intensity (e.g. incorporating short-duration high-intensity interval training (HIIT) and sprint interval training (SIT)). Whilst for health and wellbeing, the current guidelines recommend performing regular bouts of moderate-intensity exercise, totalling 150 min/week (Bull et al. 2020), high-intensity interval exercise is also now considered to be an important intervention for maintaining cardiovascular and metabolic health across the general population and in clinical conditions (Gibala 2018).

Whist it has long been established that endurance training at moderate intensities of exercise (~ 60–80% $\dot{V}$O_2_max) results in an increased capillarisation (Andersen and Henriksson 1977; Hoppeler et al. 1985; Denis et al. 1986), less is known as to the effect of higher intensity exercise on skeletal muscle capillary supply. Jensen et al. (2004) showed that HIIT (involving repeated 1 min exercise periods at 150 and 90% of $\dot{V}$O_2_peak) resulted in increases in capillarisation within the first 4 weeks of training in previously untrained subjects. Similar low-volume HIIT models used by Scribbans et al. (2014) and Tan et al. (2018) (involving 20–60 s intervals) also resulted in an increase in capillarisation after 6 weeks of training in recreationally active men and previously inactive women, respectively. Increases in capillarity were observed following 6 weeks of SIT (4–6 × 30 s maximal sprints) in sedentary males, which was as equally effective as endurance training (40–60 min cycling at ~ 65% $\dot{V}$O_2_max) (Cocks et al. 2013). A similar observation was made by Daussin et al. (2008) when comparing continuous endurance training (20–35 min at ~ 65% peak work rate) with interval training (60 s at ~ 90% peak work rate) in sedentary subjects, although the magnitude of change in capillarisation was less with interval training compared to continuous training.

Despite these observations demonstrating the potency of high-intensity interval training, it seems, however, that continued training does not provide an adequate angiogenic stimulus to induce further capillary growth. In the study by Jensen et al. (2004), after the initial 4 weeks of HIIT, a further 3 weeks of training did not promote any further increases in capillarisation, despite a progressive increase in training volume (Jensen et al. 2004). The reduced potential for HIIT to induce capillarisation was further observed by Hoier et al. (2013a) in which recreationally active participants were pre-conditioning with 4 weeks of endurance training (60 min at ~ 65% $\dot{V}$O_2_max) followed by 4 weeks of HIIT (1 min intervals at ~ 120% $\dot{V}$O_2_max), whereby no further increase in capillary growth was observed following this intensified period of training.

Further evidence for the blunting of capillary growth has been shown in already conditioned skeletal muscle. Gliemann et al. (2015b) demonstrated that 8 weeks of HIIT (1 min intervals of 10, 20 and 30 s at 90, 60 and 100% maximal running speed, respectively) in already recreationally trained runners ($\dot{V}$O_2_peak of ~ 50 mL kg min^−1^) had no impact on muscle capillarity. Similar observations were made by Mitchell et al. (2019) in which there was an absence of changes in capillarity following 4 weeks of SIT (4–7 × 30 s maximal sprints) in trained cyclists and triathletes ($\dot{V}$O_2_peak of ~ 63 mL kg min^−1^). Indeed, the trained status of the participants in the study by Mitchell et al. (2019) was reflected by the high baseline muscle capillary:fibre ratio (~ 2.9) which was almost double that of the untrained participants (~ 1.6) in the SIT study that induced a ~ 30% increase in capillary:fibre ratio (Cocks et al. 2013). Finally, Nyberg et al. (2016) reported a decline in capillary density, capillary:fibre ratio and capillary contacts in highly trained soccer players following a period of low-volume speed endurance training, consisting of 30-m sprints with passive recovery, that was in addition to their regular conditioning activities.

Thus, it seems that regardless of the intensity of exercise training, the conditioned status of the muscle is a critical determinant of the angiogenic potential. The mechanism for this is not currently known but it might be that the shear stress signal for adaptation is normalised following the initial training-induced expansion of the capillary bed. An alternative suggestion might be that the tissue itself is desensitised to the putative physiological signals (Hellsten et al. 2015). Indeed, this attenuated adaptive potential of skeletal muscle as it becomes more trained is demonstrated in the acute signalling changes during exercise. Richardson et al. (2000) initially demonstrated an attenuated VEGF mRNA response following 8 weeks endurance-type training (1 h of varied training including long, slow distance, high intensity, fartlek and time-trial type sessions). Many of the studies described above demonstrated simultaneous attenuations in the molecular signalling responses alongside the attenuated capillary growth. For example, Hoier et al. (2013a) observed that the exercise-induced increases in muscle interstitial VEGF, thought to be a critical stimulus for angiogenesis, were lower following training. A similar observation was also made by Gliemann et al. (2015b) in which muscle VEGF protein content was lower following HIIT. These observations agree with the early study by Jensen et al. (2004) which showed that the proliferative capacity of skeletal muscle endothelial cells, as indicated by the co-localisation of proliferative marker Ki-67 with CD31-positive cells, declined during the latter period of HIIT. Alongside this desensitisation, it might also be the case that there is an increase in anti-angiogenic signals with time (Olenich et al. 2013).

It is under this premise that it is necessary to progress, vary and periodise the overall exercise training programme. Nevertheless, recent work has sought to investigate methods to augment the capacity to induce capillarity, particularly in the trained population. One such method is where exercise is performed in the presence of blood flow restriction (BFR) which exposes the peripheral vasculature and exercising skeletal muscle to a distorted level of blood perfusion and oxygenation that gives rise to altered shear, hypoxic, metabolic and oxidative stress signals (Ferguson et al. 2021). Evidence is accumulating that BFR, when combined with various forms of exercise including low-load resistance exercise, low- and moderate-intensity endurance exercise, and sprint interval exercise, can augment the angiogenic signalling responses when compared to non-BFR exercise (Vissing et al. 2020; Ferguson et al. 2021; Pignanelli et al. 2021).

Although the long-term effects of training with BFR on capillary growth remain to be comprehensively studied, indirect assessment of microvascular filtration capacity indicates increased skeletal muscle capillarity after low-load resistance exercise with BFR in healthy men compared to work matched, non-BFR exercise (Evans et al. 2010; Hunt et al. 2013). Recent studies have demonstrated that low-load resistance exercise with BFR increases muscle capillarity (Bjørnsen et al. 2019; Pignanelli et al. 2020; Nielsen et al. 2020), although this is not a universal finding (Jakobsgaard et al. 2018). The contrasting results may be due to differences in training frequency and volume, cuff occlusion parameters, as well as prior training status.

The classic studies of Sundberg and colleagues had already established the potential of low-to-moderate-intensity exercise training with BFR to increase muscle capillary density (Esbjörnsson et al. 1993); however, the more contemporary use of BFR utilising occlusion cuffs during endurance-type exercise is yet to be fully explored. In a series of studies that utilised moderate-intensity interval BFR exercise training (3 sets of 3 × 2 min bouts at ~ 60–80% of maximal aerobic power), Christiansen and colleagues reported an increase in leg blood flow (Christiansen et al. 2019) alongside an improved skeletal muscle oxygen delivery and uptake (Christiansen et al. 2020) suggesting that microvascular adaptive response had taken place.

Although an augmented angiogenic gene expression was observed in response to a single bout of SIT with BFR (Taylor et al. 2016), there was no increase in any index of skeletal muscle capillarity in response to 4 weeks of SIT with BFR in the study by Mitchell et al. (2019). This perhaps may not be surprising as the subjects were relatively well-trained individuals; however, there was an indication of an increased endothelial cell proliferation following SIT plus BFR (Mitchell et al. 2019). This suggests that, even in this trained population, BFR may provide an augmented stimulus for capillary growth, although this has yet to be confirmed.

Whilst the benefit of exercise training across all intensities for cardiovascular and metabolic health is not in doubt, an expansion of the capillary bed would be beneficial in many chronic diseases. However, it seems likely that the nature of exercise training in the athletic population may simply be to maintain the already expansive capillary network. Any means of further expanding is likely to have an important role given the close correlations between capillary supply and exercise capacity (Saltin et al. 1977; Coyle et al. 1988; Mitchell et al. 2018).

## Conclusion and future directions

Exercise-induced angiogenesis is a multi-faceted physiological process, involving many biochemical mediators (such as NO and VEGF), cellular contributors (circulating angiogenic blood cells) and other paracrine signalling mechanisms (exosomes). There is a clear age-related impairment in capillary density in skeletal muscle, but this can be somewhat recovered with exercise training. It is unclear if any sex differences exist in the ability to stimulate exercise-induced angiogenesis, due to methodological issues such as small sample sizes, influence of menstrual cycle and menopause on capillarity. Data reanalysed in this review article demonstrate no difference in the angiogenic response to exercise between young men and women (Fig. 4).

Skeletal muscle angiogenesis in response to exercise training appears to be an early response to a new exercise stimulus (for both aerobic and resistance exercise), with continued improvements in capillarisation proving difficult. However, the recent observations that BFR exercise can augment angiogenic signals may prove beneficial in this instance.

## Data Availability

The datasets generated during and/or analysed in this manuscipt are not publicly available but are available from the corresponding author on reasonable request.

## Abbreviations

Ang-1: Angiopoietin 1
Ang-2: Angiopoietin 2
ATP: Adenosine triphosphate
bFGF: Basic fibroblast growth factor
CAC: Circulating angiogenic cell
CO_2_: Carbon dioxide
EC: Endothelial cells
eNOS: Endothelial nitric oxide synthase
EPC: Endothelial progenitor cell
ERR-α: Estrogen-related receptor-α
EV: Extracellular vesicles
FGF-2: Fibroblast growth factor 2
Flk-1: Vascular endothelial growth factor receptor 2
HIF-1α: Hypoxia-inducible factor-1α
HIIT: High-intensity interval training
MCP-1: Monocyte chemoattractant protein 1
miRNA: Micro RNA
MMP-2: Matrix metalloprotein 2
MMP-9: Matrix metalloprotein 9
mRNA: Messenger RNA
NAD +: Nicotinamide adenine dinucleotide
NADH: Nicotinamide adenine dinucleotide + hydrogen
nNOS: Neuronal nitric oxide synthase
NO: Nitric oxide
O_2_: Oxygen
PAD: Peripheral arterial disease
PDGF-1: Platelet-derived growth factor 1
PGC-1α: Peroxisome proliferator gamma cofactor-1α
PGC-1β: Peroxisome proliferator gamma cofactor-1β
SC: Satellite cells
SIT: Sprint interval training
SkM: Skeletal muscle
Tie-2: Tyrosine kinase with immunoglobulin-like and EGF-like domains 1
Tie-2: Tyrosine kinase with immunoglobulin-like and EGF-like domains 2
TEM: Tie-2 expressing monocyte
TIMP: Tissue inhibitor of matrix metalloproteinases
TSP-1: Thrombospondin 1
VEGF: Vascular endothelial growth factor
$\dot{V}$O_2_max: Maximum oxygen uptake
$\dot{V}$O_2_peak: Peak oxygen uptake
